# Assessing Prion Infectivity of Human Urine in Sporadic Creutzfeldt-Jakob Disease

**DOI:** 10.3201/eid1801.110589

**Published:** 2012-01

**Authors:** Silvio Notari, Liuting Qing, Maurizio Pocchiari, Ayuna Dagdanova, Kristin Hatcher, Arend Dogterom, Jose F. Groisman, Ib Bo Lumholtz, Maria Puopolo, Corinne Lasmezas, Shu G. Chen, Qingzhong Kong, Pierluigi Gambetti

**Affiliations:** Case Western Reserve University, Cleveland, Ohio, USA (S. Notari, L. Qing, A. Dagdanova, K. Hatcher, S.G. Chen, Q. Kong, P. Gambetti);; Istituto Superiore di Sanità, Rome, Italy (M. Pocchiari, M. Puopolo);; Ferring Pharmaceuticals, Hvidore, Denmark (A. Dogterom);; Instituto Massone, Buenos Aires, Argentina (J.F. Groisman);; BL Consult ApS, Copenhagen, Denmark (I.B. Lumholtz);; The Scripps Research Institute, Jupiter, Florida, USA (C. Lasmezas)

**Keywords:** prions, prions and related diseases, Creutzfeld-Jakob disease, neurodegenerative diseases, transmissible spongiform encephalopathy, infectivity, urine, TSE

## Abstract

Prion diseases are neurodegenerative conditions associated with a misfolded and infectious protein, scrapie prion protein (PrP^Sc^). PrP^Sc^ propagate prion diseases within and between species and thus pose risks to public health. Prion infectivity or PrP^Sc^ presence has been demonstrated in urine of experimentally infected animals, but there are no recent studies of urine from patients with Creutzfeldt-Jakob disease (CJD). We performed bioassays in transgenic mice expressing human PrP to assess prion infectivity in urine from patients affected by a common subtype of sporadic CJD, sCJDMM1. We tested raw urine and 100-fold concentrated and dialyzed urine and assessed the sensitivity of the bioassay along with the effect of concentration and dialysis on prion infectivity. Intracerebral inoculation of transgenic mice with urine from 3 sCJDMM1 patients failed to demonstrate prion disease transmission, indicating that prion infectivity in urine from sCJDMM1 patients is either not present or is <0.38 infectious units/mL.

## Abstract 2

Intracerebral inoculation of transgenic mice failed to demonstrate prion disease transmission.

Prion diseases, a group of neurodegenerative disorders affecting humans and animals, have received considerable attention largely because of their intriguing pathogenetic mechanism and the threat they pose to public health because of their insidious infectivity. Despite their heterogeneity, all classic prion diseases are characterized by the presence of an abnormal isoform of the normal cellular prion protein (PrP^C^), which predominantly accumulates in the central nervous system (*1*). The abnormal isoform, identified as scrapie PrP or PrP^Sc^, is thought to form from a posttranslational change in conformation of PrP^C^. Prion diseases can also be transmitted by an infectious mechanism because exogenous PrP^Sc^ can impose its conformation on to the host’s PrP^C^ through a PrP^Sc^-templated conversion process (*1*).

The most common form of human prion disease, Creutzfeldt-Jakob disease (CJD), can be sporadic, inherited, or acquired by infection; the sporadic form alone accounts for the great majority of all the cases of CJD (*2*). Following protease digestion, the unglycosylated form of PrP^Sc^ exhibits the electrophoretic mobilities of either 21 kDa or 19 kDa (*3*). These two PrP^Sc^ isoforms, named PrP^Sc^ types 1 and 2, respectively, along with the methionine/valine polymorphism at codon 129 of the PrP gene, led to the current classification of sporadic CJD (sCJD) in 5 phenotypically distinct subtypes (*2**,**3*). The sCJDMM1 subtype (affecting persons homozygous for methionine at codon 129 and carrying the PrP^Sc^ type 1) accounts for ≈70% of all cases of sCJD and unquestionably is the most prevalent type of human prion disease (*2**,**3*).

PrP^Sc^ is generally considered the major, if not the sole, component of the infectious agent in prion diseases (*1**,**4**,**5*). The presence of PrP^Sc^, prion infectivity, or both has been found in several tissues and organs outside the central nervous system in prion-affected humans and animals (*6**–**10*). These findings have caused mounting concerns regarding the risk of human transmission of the disease from a variety of sources, including the consumption of prion contaminated meat and other animal products, the use of contaminated surgical instruments and medicinal products, and the exposure to waste from infected humans and animals. In this context, body fluids such as saliva, milk, and urine have received particular attention as they may support horizontal transmission and environmental contamination, which in turn may contribute to the propagation of ovine scrapie and chronic wasting disease (CWD) of cervids. Indeed, saliva has been reported to be a source of prion in scrapie-infected sheep and CWD-infected deer (*11**–**14*), whereas milk has been found to contain prions in scrapie infected sheep (*15**,**16*).

The detection of PrP^Sc^ by immunoblotting has been previously reported in urine of prion-affected hamsters and humans (*17*). However, this observation has not been confirmed in 3 subsequent studies, which instead have suggested that the original immunoblot finding resulted from nonspecific cross-reaction either with contaminating bacterial proteins (*18*) or urinary IgG fragments (*19**,**20*). Recently, prion infectivity has been detected in urine from experimentally prion-infected animals, including hamsters (*21**,**22*), deer (*13*), and mice in association with lymphocytic nephritis (*23*). However, infectivity in urine from naturally prion-affected animals has never been reported.

It is difficult to extrapolate the animal data on urine infectivity to human urine, especially for sCJD, because this form of prion disease is believed to start spontaneously in the brain rather than being caused by exogenous infection. Nevertheless, the possibility that PrP^Sc^ is indeed present in urine of sCJD patients exists because small quantities of PrP^Sc^ have been identified in peripheral organs of sCJD patients (*6**,**7**,**10*). Furthermore, we have recently shown that normal urine contains discrete amounts of a C-terminal fragment of PrP, matching the so-called C1 fragment but not full-length PrP (*24*). Although C1 may not be a good substrate for PrP^Sc^ replication, and its conversion to PrP^Sc^ has never been reported, the possibility of conversion may not be ruled out (*25**,**26*).

The presence of prions in urine of CJD patients would obviously pose serious risks relating to the medicinal use of urine-extracted proteins, hormones, and urokinase as well as collection and disposal of patient urine. Indeed, it has recently been reported that fragments of PrP, consistent with the urine PrP present in normal urine described above, co-purify with urine-derived gonadotropins (*24**,**27*). This finding has prompted the claim that PrP^Sc^ may also co-purify with urinary gonadotropins (*27*).

With the exception of early failed attempts to transmit prion disease to rodents and nonhuman primates with urine from CJD patients (*28**,**29*), no investigation on prion infectivity of human urine has been reported. We searched for prion infectivity in urine obtained from patients with sCJDMM1, the most common form of sCJD, by bioassay that used transgenic (Tg) mice expressing human PrP (*30*).

## Materials and Methods

### Patients

Samples from 4 patients (patients 1–4) affected by typical sCJDMM1 that was histologically and immunochemically confirmed (with no clinical signs of inflammatory kidney disease) were provided by the National Prion Disease Pathology Surveillance Center. The patients were 54, 68, 60, and 69 years old with disease durations of ≈2, 2, 3, and 2 months, respectively (*2*). Approvals from the Institutional Review Board and the Institutional Animal Care and Use Committee were obtained.

### Urine

Urine samples from sCJDMM1 patients 1–3 collected 2 weeks, 1 month, and 1 week before death, and from 3 healthy controls, were concentrated 100-fold by ultrafiltration with Millipore Centricon Plus 70 (Millipore, Billerica, MA, USA) (10-kDa cutoff) by using a Beckman (Miami, FL, USA) centrifuge at 3,000 × *g*. Concentrated urine was dialyzed against phosphate-buffered saline (PBS) (4 L each time) at 4°C by using the Pierce Slide-A-Lyzer cassette (Thermo Fisher Scientific, Inc., Rockford, IL, USA) (10-kDa cutoff) with 2 additional changes over 2 days.

### Brain Homogenates and Microsomal Fractions

Brain homogenates (BH) from inoculated mice (10%, wt/vol) were prepared at 4°C in PBS cleared by centrifugation at 1, 000 × *g* for 5 min. Microsomal fractions (MF) were prepared from the frontal cortex of the sCJDMM1 patients according to Reichl et al. (*31*). BH (10%, wt/vol) in PBS were centrifuged at 700 × *g* for 10 min at 4°C. The supernatant was collected and the pellet, resuspended in PBS at 30% (wt/vol), was centrifuged as above. This supernatant, pooled with the previous one, was recentrifuged at 10,000 × *g* for 7 min at 4°C. The pellet was discarded and the supernatant was centrifuged at 100,000 × *g* for 1 h at 4°C. The final pellet, representing the MF, was resuspended in PBS at 10% (wt/vol). Transgenic mice expressed full-length human PrP-129M at wild type level in mouse PrP null background, Tg(HuPrP-129M)*Prnp^0/0^* (Tg40) (*30*).

### Effect of Concentrated and Dialyzed Urine on Prion Infectivity

MF of sCJDMM1 patient 4 was spiked in 100× concentrated and dialyzed normal urine. The sample was 10-fold serially diluted and intracerebrally inoculated into Tg40 mice.

### Determination of Infectivity Loss during Concentration and Dialysis

MF (100 µL) of sCJDMM1 patient 3 was spiked into normal urine (100 mL) either before or after 100× concentration and dialysis. Subsequently, 30 µL of the differently processed urine samples, both containing an equivalent amount of 3 µL of MF, were intracerebrally injected into Tg40 mice. The infectivity titers of the 2 differently processed MF spiked urines were compared on the basis of their mean incubation times in Tg40 mice.

### Inoculation

Thirty microliters of MF from sCJDMM1 patients 1–4 suspended in PBS or in 100× concentrated and dialyzed normal urine were intracerebrally injected into Tg40 mice (Table 1, Table 2, Table 3, Table 4). Infectivity of urine from 3 sCJDMM1 patients and 3 healthy subjects was assayed as above by inoculation of 30 µL of 100× concentrated and dialyzed urine; raw urine from sCJDMM1 patient 1 was also similarly bioassayed in 33 Tg40 mice. Mice were euthanized within 3 days of becoming symptomatic.

**Table 1:** Infectivity titers in microsomal fraction of 4 patients with sCJDMM1*

sCJDMM1 inoculum	Brain† dilution	Incubation time, d , mean ± SEM	Distribution	Prion titer, ID_50_/g tissue†
Patient 1	10^−1^	256 ± 5	7/7	3.0 × 10^6^
	10^−2^	297 ± 18	4/4	
	10^−3^	314 ± 15	5/5	
	10^−4^	339 ± 16	5/5	
	10^−5^	420, 437	2/5	
	10^−6^	>681	0/5	
Patient 2	10^−4^	382 ± 12	5/10	2.2 × 10^6^
Patient 3	10^−4^	348 ± 12	10/10	1.2 × 10^7^
Patient 4	10^−2^	267 ± 14	5/5	7.2 × 10^6^

*sCJD, sporadic Creutzfeldt-Jakob disease; ID_50_, 50% infectious dose. †Brain tissue equivalent.

**Table 2:** Prion infectivity in microsomal fraction prepared from sCJDMM1 patient 4 and suspended in PBS or 100× concentrated and dialyzed urine*

Carrier	Brain† dilution	Incubation time, d, mean ± SEM	Distribution	Prion titer, ID_50_/g tissue†
PBS‡	10^−2^	267 ± 14	5/5	7.2 × 10^6^
100× concentrated and dialyzed normal human urine	10^−1^	262 ± 4	5/5	3.3 × 10^5^
	10^−2^	281 ± 7	5/5	
	10^−3^	377 ± 35	6/6	
	10^−4^	311, 335	2/4	
	10^−5^	>793	0/6	
	10^−6^	>702	0/6	

*sCJD, sporadic Creutzfeldt-Jakob disease; PBS, phosphate-buffered saline; ID_50_, 50% infectious dose. †Brain tissue equivalent. ‡Titer was the same as calculated in Table 1.

**Table 3:** Determination of infectivity loss of MF from sCJDMM1 patient 3 spiked in normal urine, during concentration and dialysis procedures*

Inoculum	Brain† dilution	Incubation time, d, mean ± SEM	Distribution	Prion titer, ID_50_/g tissue†
MF in normal urine, then concentrated 100× and dialyzed	10^−2^	265 ± 12‡	8/9	8.2 × 10^6^
MF in 100× concentrated and dialyzed normal human urine	10^−2^	268 ± 10‡	8/8	6.9 × 10^6^

*MF, microsomal fraction; sCJD, sporadic Creutzfeldt-Jakob disease; ID_50_, 50% infectious dose. †Brain tissue equivalent. ‡p = 0.42 by *t*-test.

**Table 4:** Native prion infectivity of concentrated and dialyzed as well as raw urine from 3 sCJDMM1 patients by bioassay in Tg40 mice*

Inoculum	Incubation time, d	Distribution
100× concentrated and dialyzed urine (sCJDMM1 patient 1)	>736	0/10
100× concentrated and dialyzed (sCJDMM1 patient 2)	>788	0/10
100× concentrated and dialyzed (sCJDMM1 patient 3)	>788	0/8
100× concentrated and dialyzed (normal control 1)	>719	0/5
100× concentrated and dialyzed (normal control 2)	>756	0/5
100× concentrated and dialyzed (normal control 3)	>752	0/5
Raw urine (sCJDMM1 patient 1)	>857	0/33

*sCJD, sporadic Creutzfeldt-Jakob disease.

### Infectivity Titration

**Figure 1:**
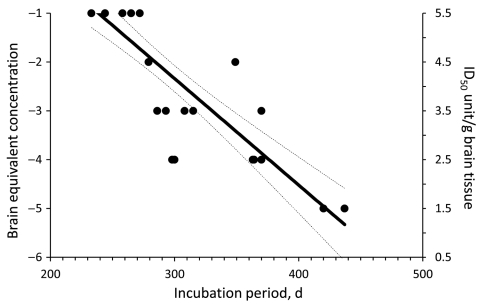
Dose-incubation period curve of brain microsomal fraction from sporadic Creutzfeldt-Jakob disease MM1 (patient 1) intracerebrally injected into Tg40 mice. Each solid circle represents the incubation time for single animal (x-axis) at different brain tissue equivalent dilution. Animals with the same incubation times have overlapping solid circles. Right y-axis is the amount of infectivity present in the inoculum at each brain tissue equivalent dilution (left y-axis). The experimental points were fitted by linear regression curve and 50% infectious dose (ID_50_) calculated by using probit-nonlinear regression analysis. Dashed lines indicate 95% confidence interval.

Infectivity in MF from sCJDMM1 patients 1 and 4, the latter spiked and diluted in 100× concentrated and dialyzed normal urine, was estimated by endpoint dilution by using probit regression analysis (STATA version 8.2 software; StataCorp LP, College Station, TX, USA). Incubation periods (Figure 1, x-axis) of each mouse injected with known infectivity titers (Figure 1, y-axis) of sCJDMM1 patient 1, were plotted and the experimental points fitted either by linear or segmental linear regression curves (GraphPad Prism version 5.0b for Macintosh; GraphPad Software, San Diego, CA, USA), and the 2 models were compared by the extra sum-of-squares F test for determining the best model fitting the experimental points. The more complicated model (segmental linear regression) did not significantly (p = 0.4033) fit the experimental points better than the simpler linear regression model. The linear regression model was therefore selected for estimating the infectivity titers in brain preparations of sCJDMM1 patients 2 and 3, and PBS spiked with MF preparation from sCJDMM1 patient 4. Mean incubation periods of animals with positive test results were interpolated to the dose-incubation-period curve of patient 1 to estimate infectivity titers.

The amount of prion infectivity that is below the threshold of detectability of our bioassay but that might be present in the urine of the sCJDMM1 patients was estimated in infectious unit (IU) per milliliter with 95% confidence interval (CI) (rather than in 50% infectious dose [ID_50_]) by assuming a Poisson distribution in the response variable (*32*). The total volume of native urine assayed in sCJDMM1 patients 1–3 combined was 84 mL when the 100-fold concentration is taken into account (0.03 mL × 10 mice each in patients 1 and 2 and 0.03 mL × 8 mice in patient 3 × 100-fold concentration). However, considering the 20-fold loss of prion infectivity caused by the use of 100-fold concentrated and dialyzed urine as carrier (see Results), the injected urine equivalents might be 20× lower, i.e., 4.2 (1.5 mL in patients 1 and2, and 1.2 mL in patient 3). Urine infectivity in patient 1 was also estimated by assuming a Poisson distribution based on the volume of raw urine bioassayed from this patient which was 0.99 mL (30 µL × 33 mice).

### Histopathologic and Prion Protein Immunohistochemical Analyses

Half brains fixed in formalin and immersed in 98% formic acid for 30 min were sliced into 4 coronal sections and processed for histologic and PrP immunohistochemical analyses (*30*). Aliquots of BH or sodium phosphotungstate (NaPTA)–precipitated samples, with or without treatment with proteinase K (PK) (specific activity 44 units/mg; Sigma Aldrich, St. Louis, MO, USA) at a concentration of 2 U/mL (45 µg/mL), were loaded onto 15% Tris-glycine sodium dodecylsulfate–polyacrylamide gels, subjected to electrophoresis, and immunoblotted with monoclonal antibody 3F4 (to PrP residues 109–112) (*9**,**33*).

## Results

### Prion Infectivity Titer of Brain Tissue from sCJDMM1

**Figure 2:**
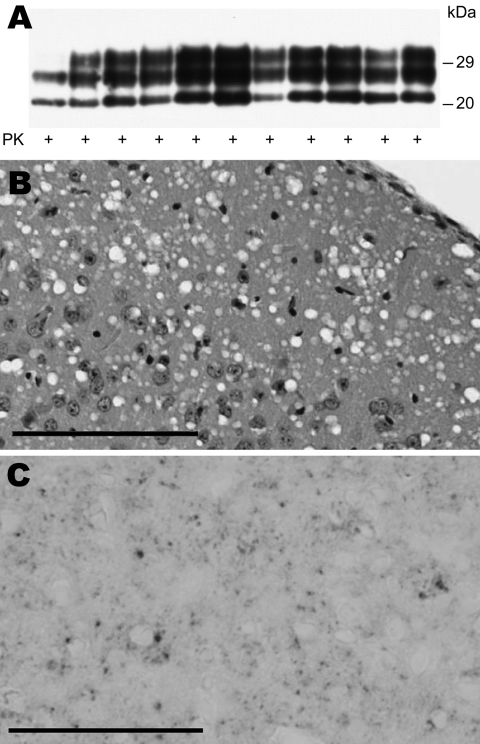
Immunochemical and histopathologic study of humanized transgenic (Tg) mice inoculated with sporadic Creutzfeldt-Jakob disease MM1 (sCJDMM1) microsomal fraction (MF). A) Immunoblot of proteinase K (PK)–resistant scrapie prion protein (PrP^Sc^) from brains of 10 Tg40 mice inoculated with MF from a patient with sCJDMM1. The inoculum sCJDMM1 MF is shown as control in the first lane. Histologic (B) and immunohistochemical (C) studies show widespread spongiform degeneration and punctate PrP^Sc^ immunostaining of the cerebral cortex from the inoculated mice. Monclonal antibody 3F4 was used for all immunostaining. Scale bar in B = 100 μm. Scale bar in C = 50 μm.

All mice inoculated with brain MF from the 4 sCJDMM1 patients showed brain histologic lesions, PrP^Sc^ deposition patterns, and immunoblot profile of PK-resistant PrP^Sc^ observed following sCJDMM1 transmission (Figure 2). The prion infectivity titer in the MF obtained from sCJDMM1 patient 1 was 3.0 × 10^6^ ID_50_ per gram of tissue equivalent as determined by endpoint dilution bioassay in Tg40 mice (Table 1; Figure 1). We then used incubation times of each mouse inoculated with different brain tissue equivalent dilutions to build up the dose-incubation period curve of Figure 1 (slope ± SE −0.022 ± 0.002; Y-intercept ± SE 10.69 ± 0.73; R^2^ 0.75; p<0.0001). Titers from brain MF of sCJDMM1 patients 2–4 were estimated by interpolating the mean incubation times of the inoculated Tg40 mice to the dose-incubation period curve of Figure 1. The results showed an infectivity titer between 2 and 12 × 10^6^ ID_50_ per gram of brain tissue equivalent (Table 1). These data indicated that the frontal cortex of all 4 sCJDMM1 patients contained high and comparable prion infectivity titers.

### Urine Concentration and Dialysis

To maximize the sensitivity of bioassay, urine samples were concentrated 100× by ultrafiltration and dialyzed against PBS before bioassay in the humanized Tg40 mice. The potential toxic effect of intracerebral injection of concentrated and dialyzed urine was assessed by injecting 3 Tg40 mice with 100× concentrated and dialyzed urine collected from a healthy subject. The inoculated mice showed no clinical signs and were euthanized at 7, 14, and 21 days postinoculation (dpi). The histologic examination revealed no lesions (data not shown). These data show that 100× concentrated and dialyzed urine is not toxic following intracerebral inoculation into Tg40 mice.

### Effect of Concentrated and Dialyzed Urine on Prion Infectivity

The effect on prion infectivity of the concentrated and dialyzed urine when used as carrier was assessed by comparing infectivity titers of MF from cerebral cortex of sCJDMM1 patient 4 diluted either in PBS or in 100× concentrated and dialyzed urine. The bioassay analyses showed that using concentrated and dialyzed urine as carrier the infectivity titer of MF was reduced ≈20-fold (1.3 log reduction) (Table 2), indicating that 100× concentrated and dialyzed urine can support prion infectivity although less efficiently than PBS.

### Effect of Concentration and Dialysis Procedures on Prion Infectivity

The possible loss of prion infectivity during concentration and dialysis procedures was assessed by bioassay. We compared the infectivity titers of brain MF from sCJDMM1 patient 3 spiked in normal urine before the concentration and dialysis procedure with the infectivity of the same amount of MF spiked in normal urine already concentrated and dialyzed (Table 3). Similar mean incubation periods of 265 days and 268 days, respectively, and prion titers of 8.2 × 10^6^ ID_50_/g and 6.9 × 10^6^ ID_50_/g, respectively, were found in the 2 differently processed samples, showing that the concentration and dialysis procedure per se had no detectable effect on infectivity.

### Bioassay of Concentrated and Dialyzed Urine and Raw Urine from sCJDMM1 Patients

**Figure 3:**
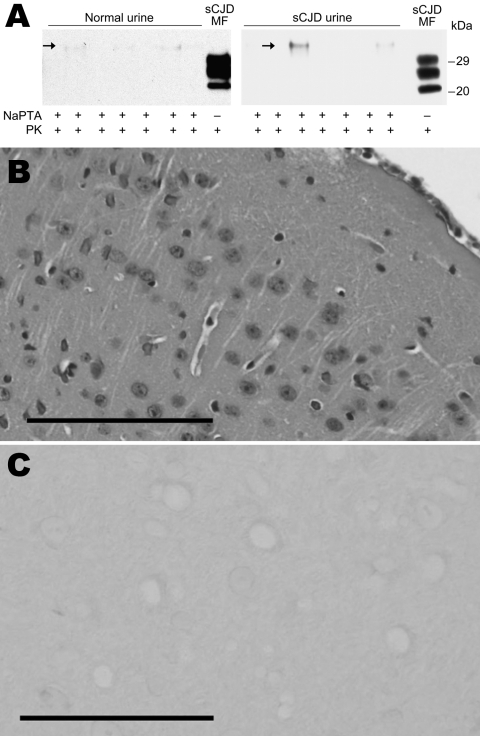
Immunochemical and histopathologic study of humanized transgenic (Tg) mice inoculated with urine from patients with sporadic Creutzfeldt-Jakob disease MM1 (sCJDMM1). A) Immunoblot of brain homogenates (BH) from Tg40 mice inoculated with 100-fold concentrated and dialyzed urine from a sCJDMM1 patient show no proteinase K (PK)-resistant scrapie prion protein (PrP^Sc^). Before immunoblotting, BH samples were treated with sodium phosphotungstate (NaPTA) to concentrate the PrP^Sc^ possibly present. BH from a Tg40 mouse inoculated with sCJDMM1 MF is shown as a positive control. A nonspecific band ≈32 kDa was detected in normal and sCJDMM1 urine (arrow). All samples were treated with PK. Histologic (B) and immunohistochemical (C) examinations show neither lesions nor abnormal PrP deposits in the cerebral cortex from urine-inoculated mice. Monoclonal antibody 3F4 was used for all immunostaining**.** Scale bar in B = 100 μm. Scale bar in C = 50 μm.

To detect the prion infectivity in urine from sCJDMM1 patients, urine samples collected from 3 end-stage sCJDMM1 patients were concentrated 100× by ultrafiltration followed by dialysis against PBS. The concentrated and dialyzed urine from the 3 donors was intracerebrally inoculated into 8–10 Tg40 mice. Over the expected normal lifespan (up to 788 dpi), no mice showed any evidence of clinical disease (Table 4). No PK-resistant PrP^Sc^ was detected by Western blot, even after enrichment with NaPTA precipitation (Figure 3, panel A). Results of histopathologic and PrP immunohistochemical examinations also were negative (Figure 3, panel B).

On the basis of these negative results, we estimate that prion infectivity in urine of sCJDMM1 patients to be 0 IU/total volume of inoculated urine, which by Poisson distribution is less than 0.37 IU for patients 1 and 2 and 0–0.46 IU for patient 3 (upper limit of 95% CI). However, if we take into account the 100× concentration of urine and the finding that 100× concentrated and dialysized urine may reduce prion infectivity by ≈20-fold (Table 2), the estimates of the highest prion titers possible (i.e., the upper 95% CI) of urine in sCJDMM1 are 0.24 IU/mL for patients 1 and 2 and 0.38 IU/mL for patient 3 (see Materials and Methods).

To directly assess the prion infectivity in urine without concentration and dialysis, we inoculated 33 Tg40 mice with raw urine from sCJDMM1 patient 1. No inoculated mice showed clinical signs of prion disease over their normal lifespan (up to 857 dpi) (Table 4). Similarly, histopathologic and PrP immunohistochemical examination were negative, and no PK-resistant PrP^Sc^ was detected by Western blot in the brain even after PrP^Sc^ enrichment with NaPTA (data not shown). Thus, the estimated infectivity titer in raw urine of sCJDMM1 by Poisson distribution is 0–0.11 IU/mL (95% CI), not dissimilar to the value (0–0.24) obtained with the same urine after concentration and dialysis.

## Discussion

The present study demonstrates that the urine from patients affected by advanced sCJDMM1, the most common sCJD subtype that alone accounts for ≈60% of all human prion diseases, contains either no prion infectivity or an infectivity titer that is below the detection limit of our bioassays. The bioassays were done in Tg mice expressing human PrP-129M (Tg40) following inoculation with urine obtained from patients with sCJDMM1 and a variety of positive and negative controls. In limit dilution experiments, Tg40 mice inoculated with MF preparations obtained from the brains of 3 urine donors with sCJDMM1 had prion disease develop at up to 10^5^ or 10^4^ dilutions of the brain tissue equivalent depending on whether the MF preparations were inoculated directly or after spiking into concentrated and dialyzed normal human urine.

To enhance the sensitivity of our system, urine samples were concentrated and dialyzed before inoculation. Similar procedures have been used in all the previous studies on prion infectivity of urine (*13**,**22**,**23*), except for the study by Gregori et al. (*21*). Our procedure is similar to that used by Seeger et al., who reported the detection of prion infectivity in urine from scrapie inoculated mice affected by nephritis (*23*). Although we demonstrated in the spiking experiment with MF from sCJDMM1 that the 100× concentration and dialysis procedure did not cause infectivity loss (Table 3), the infectivity of the prion-spiked preparation decreased 20-fold when 100× concentrated and dialyzed urine was used as carrier compared with PBS. On the basis of these findings, we estimated that if prion infectivity is present at all in sCJDMM1 urine, it is at most 0.38 IU/mL if the 20-fold infectivity loss is factored in. Because the nature of the potential PrP^Sc^ in urine from CJD patients is not known, this urine PrP^Sc^ species might show even higher loss of infectivity in the concentrated and dialyzed urine carrier than the brain PrP^Sc^ preparations used in the spiking experiments. To address this concern, we inoculated 33 Tg40 mice with raw urine from one of the 3 donors with sCJDMM1. No recipient mice showed evidence of prion disease suggesting an infectivity ranging from 0.0 and 0.11 IU/per mL (upper limit of 95% CI) as estimated by the Poisson distribution.

Although asymptomatic disease in recipient mice associated with NaPTA- undetectable minute amounts of PrP^Sc^ cannot be excluded, our inability to detect prion infectivity in human urine of patients with sCJDMM1 differs from several recent experimental studies on urine of prion-affected animals. Low prion infectivity has been demonstrated in urine from scrapie-infected hamsters (*21**,**22*), CWD-infected deer (*13*), and in scrapie-infected mice affected by lymphocytic nephritis. In the last study, however, no urine infectivity was found in non-nephritic mice (*23*). Three additional studies have demonstrated the presence of PrP^Sc^ in urine from scrapie-infected hamsters and CWD-infected deer using protein misfolding cyclic amplification (PMCA) (*13**,**34**,**35*). However, this highly sensitive procedure can detect prion concentrations below the level of detectability of bioassays.

The most likely explanation for the discrepancy between our negative results on human urine and the positive findings by bioassay in urine from animals resides in the different locale and mode of formation of the prion agents. In all the published animal experiments, including bioassays and PMCA, the prion disease was induced by intracerebral or oral administration of exogenous prions, whereas we examined urine infectivity in a naturally occurring sporadic human prion disease. In exogenously acquired prion diseases, PrP^Sc^ is much more likely to be widely present in nonneural peripheral organs, including blood, kidney, and bladder, which is likely the result of early exposure of peripheral organs to the inoculated prions. In sCJD, which is believed to occur spontaneously in the brain (rather than being acquired by infection from exogenous prions), only minute amounts of PrP^Sc^ have been detected in a few nonneural peripheral organs and tissues such as skeletal muscle and spleen (*6**–**8**,**10*). In contrast, in variant CJD (vCJD), the form of CJD acquired by consumption of BSE-infected beef, the spread of PrP^Sc^ to peripheral organs is much wider and typically involves lymph nodes, tonsil, spleen, portions of the intestinal tract, and the skeletal muscle (*7**,**10**,**36**,**37*), as well as kidney and other organs (*9*). These considerations indicate that vCJD (not sCJD) in principle is more similar to the exogenously acquired animal prion diseases that have been used to study prion infectivity in urine. Therefore, vCJD urine that is more likely to contain prion infectivity should be tested by PMCA or bioassay. However, the reported infectivity of animal urine might also result, at least in some instances, from contamination with feces.

Recent data have proved that feces from hamsters infected with scrapie by the oral route and to a lesser extent through intracerebral and intraperitoneal inoculation, contain a discrete amount of PrP^Sc^ and prion infectivity (*38**,**39*). Infectivity has been demonstrated also in feces from deer orally infected by CWD (*40*). In hamsters and mice, metabolic cages were used for urine collection, a method in which cross-contamination by feces may actually occur. However, prion infectivity was also demonstrated in urine from CWD-infected deer from which urine collection could easily be performed by catheterization (although this procedure was not mentioned by the authors) (*13*).

Although additional studies are still needed to determine whether minute amounts of prion infectivity or PrP^Sc^ are present in urine from patients with sCJD and to assess the presence of infectious prion in urine from patients with every other form and subtype of human prion diseases, our study shows that urine from patients with sCJDMM1, the most common subtype of sCJD, does not contain prion infectivity detectable by our bioassay and suggests that no significant prionuria occurs in this common subtype of human prion disease.
